# Cellular Prion Protein (PrP^c^) and Hypoxia: True to Each Other in Good Times and in Bad, in Sickness, and in Health

**DOI:** 10.3389/fncel.2016.00292

**Published:** 2016-12-19

**Authors:** Sanja Ramljak, Holger Herlyn, Inga Zerr

**Affiliations:** ^1^Sciema UG, MainzGermany; ^2^Institute of Anthropology, Johannes Gutenberg University of MainzMainz, Germany; ^3^Department of Neurology, University Medical Center Göttingen and German Center for Neurodegenerative DiseasesGöttingen, Germany

**Keywords:** PrP^c^, hypoxia, glycolysis, neuroprotection, cancer

## Abstract

The cellular prion protein (PrP^c^) and hypoxia appear to be tightly intertwined. Beneficial effects of PrP^c^ on neuronal survival under hypoxic conditions such as focal cerebral ischemia are strongly supported. Conversely, increasing evidence indicates detrimental effects of increased PrP^c^ expression on cancer progression, another condition accompanied by low oxygen tensions. A switch between anaerobic and aerobic metabolism characterizes both conditions. A cellular process that might unite both is glycolysis. Putative role of PrP^c^ in stimulation of glycolysis in times of need is indeed thought provoking. A significance of astrocytic PrP^c^ expression for neuronal survival under hypoxic conditions and possible association of PrP^c^ with the astrocyte-neuron lactate shuttle is considered. We posit PrP^c^-induced lactate production via transactivation of lactate dehydrogenase A by hypoxia inducible factor 1α as an important factor for survival of both neurons and tumor cells in hypoxic microenvironment. Concomitantly, we discuss a cross-talk between Wnt/β-catenin and PI3K/Akt signaling pathways in executing PrP^c^-induced activation of glycolysis. Finally, we would like to emphasize that we see a great potential in joining expertise from both fields, neuroscience and cancer research in revealing the mechanisms underlying hypoxia-related pathologies. PrP^c^ may prove focal point for future research.

## Adaptation To Hypoxia

What appears ordinary today for most of the time of the Earth’s history was not: free oxygen. Actually, both aquatic and terrestrial environments were widely devoid of free oxygen for thousands of millions of years. With appearance of photosynthesis about 3.5–3.2 billion years ago (Blankenship, 2010), oxygen was for the first time produced in considerable amounts. Yet, this early oxygen was widely consumed for further hundreds of millions of years through precipitation of Fe^2+^ ions and the formation of ferrous sulfides. Only after a steady-state between the influx of Fe^2+^ from the continents and the precipitation in the oceans was reached, about 2 billion years ago, free oxygen could be enriched in noteworthy amounts (Martin and Russell, 2003). This short survey illustrates that early heterotrophs had to sustain eons of oxygen deficiency (anoxia) and limited oxygen availability (hypoxia).

Glycolytic enzymes that capacitate endurance of low oxygen tensions most likely arose some 2 billion years before the emergence of first oxygen-consuming species (Webster, 2003). Indeed these enzymes are evolutionary highly conserved (Martin and Russell, 2003; Webster, 2003). Although vertebrates are generally regarded as highly oxygen-reliant they can efficiently switch from aerobic (oxidative phosphorylation) to “ancestral” anaerobic (anaerobic glycolysis) energy production when oxygen falls under the critical mark (Nilsson and Renshaw, 2004; Jackson and Ultsch, 2010). An extreme example represents a freshwater turtle (*Trachemys scripta elegans*) which can withstand 24 h of anoxia and subsequent re-oxygenation without any apparent loss of neurons (Kesaraju et al., 2009). On the contrary, only short anoxia is sufficient to cause flat electroencephalogram in the human brain (Rossen et al., 1943). Hence, the ability to sense oxygen deprivation is vital to the survival of all aerobic organisms (Lutz and Prentice, 2002).

To survive every healthy cell has to maintain abundant adenosine triphosphate (ATP) levels and regulated metabolic depression, i.e., hypometabolism seems to be the key to survival under conditions of low oxygen (Boutilier, 2001). Consequently, when ATP levels drop reallocation of cell’s energy supplies between critical and non-critical ATP-consuming processes becomes pivotal. It seems that ATP-driven processes are ordered in hierarchy with protein and DNA/RNA synthesis ranked as low priority processes, therefore inhibited first, and fueling of ATP-dependent membrane pumps such as Na^+^/K^+^ ATPase and Ca^2+^ cycling having the highest operating priority (Buttgereit and Brand, 1995). Keeping the latter processes functional is fundamental within the central nervous system (CNS), especially when oxygen supply is sparse.

## Effects Of Hypoxia On Neurons And Astrocytes

Within the CNS, neurons are the most susceptible cell type in respect to oxygen deprivation. This is an outcome of their high aerobic metabolism. Approximately 50% of neuronal energy expenditure is committed to preserving high priority processes: ionic gradients and fluxes (Hansen, 1985). As a result, when neuronal ATP production fails to meet energy demands mandatory for sustaining ionic and osmotic equilibrium neuronal cell death follows.

In contrast to neurons, astrocytes possess glycogen stores (Magistretti, 2008) and can increase their glycolytic capacity when oxygen supply is inadequate (anaerobic glycolysis) and ATP generation via oxidative phosphorylation flawed. They are also able to increase glycolysis when oxygen levels are adequate (aerobic glycolysis).

Hence, astrocytes can withstand hypoxia without major morphological changes up to 12 h (Yu et al., 1989). An increase in glycolytic capacity of astrocytes is put into action via up-regulation of anaerobic isoforms of glycolytic enzymes such as lactate dehydrogenase A (LDH-A; Marrif and Juurlink, 1999). In addition, astrocytes are also efficient in decreasing ATP consumption when oxygen- and glucose-deprived (Yager et al., 1994). All these traits of astrocytic adaptation to low oxygen tensions presumably contribute to their role in safeguarding neurons from detrimental effects of anoxia and hypoxia (Vibulsreth et al., 1987; Imuta et al., 2007). Previous studies have demonstrated that after ischemic insult neurons fail to survive if neighboring astrocytes are not viable (Takano et al., 2009). Therefore, one can deduce that oxygen deprivation promotes release of certain astrocytic metabolic products, which are crucial for preserving neuronal vitality.

## PrP^c^-Mediated Neuroprotection Against Hypoxia

Oxidative damage is a common denominator of neurodegenerative disorders (reviewed in Zhang et al., 2011). In prion diseases, which are characterized by neuronal loss and astrogliosis (Belay, 1999), the failure in antioxidant defense seems to be crucial (Brown, 2005). The PrP^c^, which plays a central role in prion diseases, manifests antioxidant properties (Steele et al., 2007) which are obstructed by its conversion into a misfolded, disease-specific isoform (PrP^sc^).

Despite the fact that PrP^c^ is highly conserved across mammals (Schätzl et al., 1995), PrP^c^ knockout mice (Prnp^-/-^) show only subtle phenotypes under physiological conditions. However, when cellular energy requirements increase, as under different stress conditions, PrP^c^ presence becomes critical to the survival (Steele et al., 2007). As PrP^c^ expression level is the highest within the CNS, its functions at this site are presumably of uppermost relevance. Actually, one of the best-supported PrP^c^ functions so far is neuroprotection against hypoxic damage (McLennan et al., 2004; Weise et al., 2004; Mitteregger et al., 2007; Doeppner et al., 2015), implying PrP^c^ capacity for sensing and adequately responding to oxygen deprivation. Thus, PrP^c^ expression is up-regulated following cerebral ischemia, and wild-type (WT) mice display significantly smaller infarct volumes as compared to Prnp^-/-^ mice (McLennan et al., 2004; Weise et al., 2004; Mitteregger et al., 2007). Moreover, considerably increased long-term neuroprotection, neurogenesis and angiogenesis was reported in the ischemic brains of PrP^c^-overexpressing (Prnp^+/+^) vs. WT and Prnp^-/-^mice, accenting the importance of elevated PrP^c^ levels in preventing hypoxia-induced neuronal damage (Doeppner et al., 2015). In other words, it appears that a metabolic switch between oxidative-independent and oxidative-dependent metabolism during hypoxia and subsequent re-oxygenation cannot be efficiently executed when PrP^c^ is absent.

Prior study employing astrocyte-neuron co-cultures showed that PrP^c^ expression in astrocytes is fundamental for neuronal differentiation and survival (Lima et al., 2007). Moreover, astrocytic PrP^c^ expression appears to be important for reduction of hydrogen peroxide toxicity (Bertuchi et al., 2012), a reactive oxygen species whose production in mammalian cells is stimulated by hypoxia (Moller, 2001).

Considering that:

(i) astrocytes predominantly rely on glycolytic metabolism and can successfully endure hypoxic episodes;
(ii) astrocytes protect neuronal integrity from different insults;
(iii) astrocytic PrP^c^ expression is pertinent to neuronal survival and
(iv) PrP^c^ confers neuroprotection in a model of focal cerebral ischemia,

it is conceivable that astrocytic PrP^c^ expression may have a considerable influence on a favorable neurologic outcome under hypoxic conditions. Yet, which molecular scenario could support this concept?

## PrP^c^, Glycolysis, And The Astrocyte-Neuron Lactate Shuttle

Pellerin and Magistretti (1994) proposed a so-called astrocyte-neuron lactate shuttle (ANLS) model postulating that neuronal activity increases extracellular levels of glutamate, which is readily absorbed by astrocytes resulting in stimulation of astrocytic glycolysis and lactate production. Subsequently, lactate is shuttled from astrocytes to neurons via monocarboxylate transporters (MCTs) and further utilized by neurons for oxidative-and non-oxidative-derived ATP production (Bélanger et al., 2011).

Lactate is produced in the last step of the glycolytic pathway by reduction of pyruvate and concomitant oxidation of nicotinamide adenine dinucleotide (NADH) to NAD^+^ in a reaction catalyzed by the LDH-A isoform, when oxygen supply is low. In the opposite direction, lactate is converted to pyruvate by the LDH-B isoform (Le et al., 2010). Favorable effects of lactate on neuronal survival following hypoxia/ischemia are meanwhile widely recognized (Schurr et al., 1988, 1997, 2001; Berthet et al., 2009). Recently, we demonstrated that PrP^c^ markedly enhances expression of both LDH-A and LDH-B isoenzymes after hypoxia/ischemia in WT primary cortical neurons and in WT ischemic brains as compared to PrP^c^ knockout counterparts (Ramljak et al., 2015). Besides, expression of the LDH-A was significantly elevated upon transfection of Prnp^0/0^ cells with the vector bearing a cDNA encoding human *PRNP* (Ramljak et al., 2008). Additionally, LDH-A was not only identified as a PrP^c^ interactor protein, but also as an interactor of Doppel and Shadoo, two mammalian PrP^c^ paralogs (Watts et al., 2009). Earlier study investigating cellular distribution of the LDH isoenzymes in the hippocampus and occipital cortex of the human brain demonstrated a marked enrichment of LDH-A in astrocytes as compared to neurons (Bittar et al., 1996). Therefore, in view of ANLS it would be interesting to elucidate the role that presence/absence of PrP^c^ in astrocytes might have on LDH-A expression level/activity, lactate trafficking from astrocytes to neurons and ultimately on neuronal survival under hypoxic conditions.

## Dual Roles Of PrP^c^ In Hypoxia: Neuroprotection vs. Tumor Progression

Promoter region of the LDH-A possesses hypoxia-responsive element (HRE) which is trans-activated under hypoxic conditions by the transcription factor hypoxia-inducible factor 1 alpha (HIF-1α; Semenza et al., 1996). HIF-1 α is one of the two subunits of hypoxia-inducible factor 1 (HIF-1) transcription complex which assimilates information on oxygen availability and cellular redox homeostasis. Stabilization of HIF-1α enables adaptive response to hypoxia and other stress conditions (Semenza, 2000; Dery et al., 2005). Thus, stabilization of HIF-1α protects astrocytes from glutamate-induced damage during severe hypoxia (Badawi et al., 2012). On the contrary, in oxygenated cells, HIF-1α is rapidly degraded via ubiquitin-proteasome pathway (Huang et al., 1998). Expression of HIF-1 target genes, such as for instance LDH-A, correlate with the levels of HIF-1 α (Ke and Costa, 2006). Strikingly, HIF-1α expression is significantly decreased in Prnp^-/-^ and increased in Prnp^+/+^ mice at 24 h post-stroke (Doeppner et al., 2015) suggesting that PrP^c^ might exert its neuroprotective effects against hypoxic damage *in vivo* via direct or indirect regulation of HIF-1α and hence LDH-A/lactate.

Kleene et al. (2007) demonstrated that PrP^c^ is involved in regulation of lactate transport of astrocytes via MCT1 in conjunction with Na^+^/K^+^ ATP-ase and basigin. Astrocytes generally express MCT1 and MCT4 isoforms, engaged in lactate release, whereas neurons predominantly express MCT2 isoform, which facilitates lactate uptake (Dimmer et al., 2000; Pellerin et al., 2005; Rosafio and Pellerin, 2014). Interestingly, transient overexpression of PrP^c^ in HEK293 cells enhanced MCT1 expression under normoxic conditions (Ramljak et al., 2015). Accordingly, *in vivo* neurochemical profiling in 12 month old WT and Prnp^-/-^ mice under normoxic conditions revealed 100% increase in lactate content in the hippocampus and cerebellum of Prnp^-/-^ mice (Cudalbu et al., 2015) indicating impaired regulation of lactate in Prnp^-/-^ mice.

To the best of our knowledge so far no report considered the presence of two highly conserved early growth response -1 (EGR-1) consensus binding motifs (5′-GCG(T/G)GGGCG-3′) separated by only 15 bases between introns 1 and 2 of the human *PRNP* gene. These emerged at least 29.1 million years ago in the common stem lineage of extant Catherrini, as determined by own sequence screening (see **Table 1** for accession numbers). Binding of Egr-1 to a conserved intron sequence and consecutive regulation of gene expression has been demonstrated in mouse motor spiny neurons (Keilani et al., 2012). Egr-1 is a transcription factor that is rapidly induced by hypoxia, can directly bind to HIF-1α promoter region and *trans*-activate it (Sperandio et al., 2009), but it can also function independently of HIF-1 α (Yan et al., 1999).

**Table 1:** *EGR-1* motif in intron 1/2 of the *PRNP* gene.

	5′-GCG(T/G)GGGCG-3′	
Species abbreviation	Number of motifs	Accession numbers
*Homo sapiens*	2	ENST00000379440
*Pan troglodytes*	2	ENSPTRT00000024563
*Gorilla gorilla*	2	ENSGGOT00000008115
*Pongo abelii*	0	ENSPPYT00000012541
*Nomascus leucogenys*	2	ENSNLET00000009813
*Macaca mulatta*	2	ENSMMUT00000028037
*Papio anubis*	2	ENSPANT00000012376
*Chlorocebus sabaeus*	2	ENSCSAT00000018848
*Callithrix jacchus*	0	ENSCJAT00000041793
*Tarsius syrichta*	0	ENSTSYT00000012169
*Mus musculus*	0	ENSMUST00000091288

*Accession numbers obtained from ENSEMBL.*

Notably, studies performed on mouse brains suggest that prion diseases deregulate several microRNAs (miRNAs) and one of the gene promotors that were cognate to these miRNAs is Egr-1 (Shapshak, 2013). A so-called neurotoxic peptide PrP(106-126), broadly used as a model of neurotoxicity in prion diseases, induced Egr-1 synthesis in primary cortical neurons just 30 min after the treatment (Gavín et al., 2005) suggesting a hypoxic cellular environment. Furthermore, Seo et al. (2010) showed that low oxygen conditions protect neuroblastoma cells from neurotoxicity of PrP(106-126) by activating Akt signaling pathway and connote an involvement of hypoxia in prion-induced neuronal damage/disease. PrP(106-126) propels aggregation of endogenous PrP^c^ to an amyloidogenic form and shares several properties with the disease-causing PrP^sc^ isoform (Singh et al., 2002).

Intriguingly, distinct protein modifications and formation of detergent-insoluble protein aggregates experimentally induced by proteasome inhibition are oxygen-requiring processes that may be prevented when cells are incubated at 3% instead of 21% oxygen (Demasi and Davies, 2003). Many lines of evidence point to the deficits in cellular protein quality control and hence ubiquitin-proteasome system as central to the pathogenesis of neurodegenerative diseases (Takalo et al., 2013). Therefore, one can conclude that normoxic conditions would favor further formation of aggregates in the brains of individuals affected by neurodegenerative disorders. Contrariwise this finding suggests that hypoxia might be as well regarded as a “survival process” during which cellular machinery maintains only functions of the highest priority (protein synthesis is a low priority process!) in order to survive and concurrently prevent further formation of protein aggregates.

Both Egr-1 and HIF-1α have been associated with neurodegenerative diseases:

(i) Egr-1 is up-regulated in brains of Alzheimer disease patients and regulates transcription of genes involved in synaptic plasticity processes, in particular maintenance of long-term potentiation (Jones et al., 2001; Gómez Ravetti et al., 2010; Lu et al., 2011).
(ii) Increasing HIF-1 activity has been put forward as a potential strategy to alleviate the pathogenesis of Alzheimer’s and other neurodegenerative disorders (Zhang et al., 2011).

A recent study demonstrated that neuronal cells exposed to a highly neurotoxic monomeric misfolded prion protein (TPrP) exhibited profound decline of NAD^+^ levels followed by diminished ATP production. Neuronal death induced by TPrP could be completely rescued *in vitro* and *in vivo* by supplying NAD^+^ (Zhou et al., 2015). Primary astrocytes subjected to TPrP were not prone to TPrP-mediated toxicity and exhibited even increased NAD^+^ levels (Zhou et al., 2015). As cytosolic regeneration of NAD^+^ by LDH-A is necessary for glycolysis to carry on it would be highly interesting to verify if the treatment with TPrP renders the cellular environment hypoxic. It is recognized that diminishing NAD^+^ levels induce pseudohypoxia by disturbing nuclear-mitochondrial communication during aging (Gomes et al., 2013).

In any case, considering a role of putative synergistic networking between EGR-1-PrP^c^-HIF-1α-LDH-A under conditions of low oxygen tensions definitely deserves further attention.

Intriguingly, all four members of the above-suggested networking are in one way or another tied to another hypoxia-related disorder: cancer.

(i) EGR-1 directly targets HIF-1 α in hypoxic prostate cancer cells (Sperandio et al., 2009);
(ii) elevated HIF-1 α expression levels are linked to increased risk of mortality in different types of human cancers such as colon, breast, stomach, and other cancer types (Semenza, 2010);
(iii) HIF-1 α activates expression of *LDH-A* (Semenza et al., 1996);
(iv) inhibition of LDH-A inhibits tumor progression (Le et al., 2010);
(v) *PRNP* was proved as a prognostic indicator in patients with recurrent colorectal cancers (Antonacopoulou et al., 2010);
(vi) PrP^c^ has a potential as a biomarker of poor prognosis in pancreas ductal adenocarcinoma patients (Sy et al., 2011–2012);
(vii) PrP^c^-overexpression advances invasive and metastatic features of gastric cancer cell lines (Pan et al., 2006; Liang et al., 2007; Wang et al., 2011) and
(viii) PrP^c^-overexpression was detected in 90% of prostate tumor biopsies (Yang et al., 2014).

Lately, tumor necrosis factor (TNF)-related apoptosis-inducing ligand (TRAIL) has been identified as relevant for PrP^c^-mediated survival of cancer cells. Thus, increase in PrP^c^ expression under hypoxic conditions in human colon carcinoma HCT116 cell line was accompanied with concurrent downregulation of TRAIL (Park et al., 2015). Conversely, down-regulation of PrP^c^ increased TRAIL-induced apoptosis under same experimental conditions (Park et al., 2015). Remarkably, an up-regulation of EGR-1 has also been shown to act as a brake on TRAIL expression (Balzarolo et al., 2013). TRAIL’s ability to selectively induce apoptosis in cancer but not in normal cells is well recognized (Wu, 2009). Considering their effect on TRAIL expression, blocking PrP^c^, and/or EGR-1 should be further investigated as potentially useful anticancer treatment. Moreover, activation of phosphatidylinositol 3 kinase (PI3K)/Akt survival pathway seems to be critical to TRAIL resistance in human cancer cells whereas its inhibition sensitizes resistant cancer cells to TRAIL (Xu et al., 2010). PrP^c^ is known to modulate PI3K/Akt pathway (Vassallo et al., 2005; Weise et al., 2006).

## Cross-Talk Between Wnt/β-Catenin And Pi3K/Akt Signaling Pathways Under Low Oxygen Tensions

**FIGURE 1:**
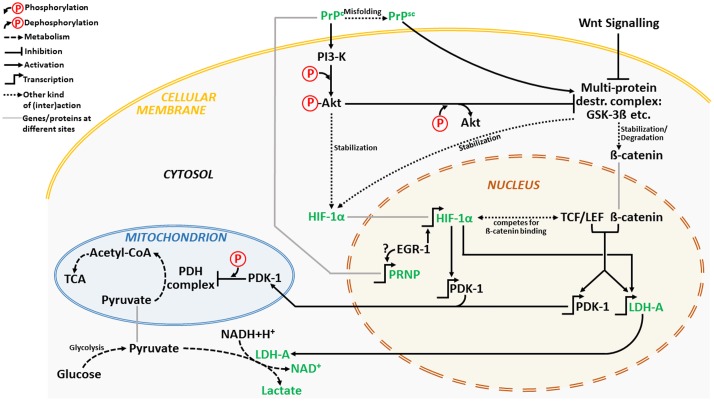
**A simplified schematic depiction of a hypothetic PrP^c^-induced signaling cross-talk between PI3K/Akt and Wnt/β-catenin pathway under hypoxic conditions.** The major players are depicted in green. For clarification, please see the Section “Cross-Talk between Wnt/β-Catenin and PI3K/Akt Signaling Pathways under Low Oxygen Tensions.”

We propose a cross-talk between Wnt/β-catenin and PI3K/Akt pathways as underlying PrP^c^-mediated survival under low oxygen tensions (**Figure 1**).

Cellular prion protein can activate anti-apoptotic PI3K/Akt pathway (Vassallo et al., 2005). Conversely, its deletion impairs the PI3K/Akt pathway by reducing phospho-Akt expression (Weise et al., 2006). Activation of PI3K/Akt pathway seems necessary for HIF-1α stabilization early during hypoxia (Mottet et al., 2003). Besides, inhibition of glycogen synthase kinase-3β (GSK-3β) activity by phospho-Akt leads to stabilization of HIF-1α and increased HIF-1 transcriptional activity (Mottet et al., 2003) (**Figure 1**).

GSK-3βis a component of the multiprotein destruction complex, a part of the Wnt/β-catenin signaling pathway (MacDonald et al., 2009) which seems pertinent for a cross-talk between the both pathways. Inhibition of GSK-3β activity by phospho-Akt stabilizes β-catenin which in turn together with TCF/LEF transcription factor promotes transcription of Wnt target genes such as: pyruvate dehydrogenase kinase 1 (PDK-1) and LDH. Recently, Wnt/β-catenin signaling was linked to activation of glycolysis in colon cancer via targeting of PDK-1 (Pate et al., 2014). Furthermore, direct targeting of PDK-1 by HIF-1 results in suppression of mitochondrial function by limiting pyruvate entry into the tricarboxylic acid (TCA) cycle (Kim et al., 2006; Papandreou et al., 2006). This kinase phosphorylates and switches off mitochondrial pyruvate dehydrogenase (PDH) complex (Roche et al., 2001) so that the conversion of pyruvate to acetyl-CoA is inhibited and conversion of pyruvate to lactate favored. Intriguingly, Wnt is also capable of enhancing LDH activity thus additionally fostering glycolysis (Chafey et al., 2009).

Cellular prion protein appears to interact with β-catenin and up-regulate transcriptional activity of the β-catenin/TCF complex (Besnier et al., 2015). Moreover, Wnt/β-catenin signaling is impaired in mice infected with scrapie agents with markedly reduced levels of phospho-GSK-3β leading to its enhanced activity (Sun et al., 2015) and degradation of β-catenin. In addition, dysfunctional PI3K-Akt-GSK-3 pathway is common in models of prion diseases (Simon et al., 2014).

If the hypothetic cross-talk between Wnt/β-catenin and PI3K/Akt pathway holds true then the interesting question would be: can PrP^sc^ mice develop cancer?

In summary, it only seems like PrP^c^ has two sides: a “good” one – if not pivotal – for neuroprotection against oxidative stress such as hypoxia and a “bad” one promoting invasiveness of different cancer types. However, these are only two sides of the same medal called: SURVIVAL.

## Author Contributions

SR developed the concept and wrote the manuscript; HH analyzed the data and wrote the manuscript; IZ wrote the manuscript.

## Conflict of Interest Statement

The authors declare that the research was conducted in the absence of any commercial or financial relationships that could be construed as a potential conflict of interest.
